# The Trajectories of Olfactory Dysfunction from the First to the Omicron Wave: Are We Getting over it?

**DOI:** 10.3390/pathogens12010010

**Published:** 2022-12-21

**Authors:** Luigi Angelo Vaira, Jérome R. Lechien, Giacomo De Riu, Sven Saussez

**Affiliations:** 1 Maxillofacial Surgery Operative Unit, Department of Medicine, Surgery and Pharmacy, University of Sassari, 07100 Sassari, Italy; 2 Biomedical Science Department, PhD School of Biomedical Science, University of Sassari, 07100 Sassari, Italy; 3 Department of Human and Experimental Oncology, Faculty of Medicine UMONS Research Institute for Health Sciences and Technology, University of Mons (UMons), B7000 Mons, Belgium; 4 Department of Otolaryngology-Head Neck Surgery, Elsan Polyclinic of Poitiers, 86000 Poitiers, France

It has now been two years since the publication in *Pathogens* of our European multicenter study on the prevalence of olfactory dysfunctions (OD) during COVID-19 [1]. To date, with 774 patients, this is the largest psychophysical study ever published. The study provided a clear picture of the burden of OD during SARS-CoV-2 infection affecting more than 60% of patients during the first two pandemic waves. These data have been confirmed by numerous other authors around the world [2,3,4,5,6]. 

It was found was that 5 to 40% of these patients developed persistent OD which is now recognized as one of the most frequent symptoms of long-COVID-19 [7,8,9,10,11,12,13]. A severe and persistent reduction in smell has deleterious effects on the quality of life of patients who are propelled towards social isolation and depression [14,15,16] and exposed to environmental hazards. For this reason, researchers’ efforts are now turning to finding risk factors for the development of persistent OD [17,18,19,20,21,22,23,24] and numerous therapeutic trials are underway around the world [25,26,27,28,29,30,31,32].

Recently, some good news has come from the clinical data of patients infected with the Omicron variant which now represents almost all cases. In these patients, the prevalence of OD during infection markedly decreased compared to the previous variants, ranging between 1 and 34% [33,34,35]. Curiously, the Omicron variant has been shown to contain the D614G mutation which has been related to SARS-CoV-2’s ability to induce OD [36]. It is possible that the lower prevalence of OD is at least in part related to a more organized and effective IgA-mediated immune response of the olfactory epithelium of immunized subjects [37]. However, this is certainly not the only reason since, during the previous pandemic waves, OD proved to be a frequent clinical finding even in vaccinated individuals [38] and reinfections [39]. It is therefore probable that factors related to the virus are also implicated: lower pathogenicity [40,41], lower ability to enter cells of the olfactory epithelium through ACE-2 receptors [42,43], lower solubility in nasal mucus [44].

However, there are still no studies with long-term follow-up data to evidence whether this reduced prevalence corresponds to a lower rate of persistent OD. From the first 6 months of collected data, currently being published, the rate of persistent OD in Omicron patients is low and does not show significant differences compared to the general population. Good news that may be a light at the end of the tunnel for those who have found themselves overwhelmed by patients with persistent OD.
